# The case for considering volar skin in a “separate status” for wound healing

**DOI:** 10.3389/fmed.2023.1156828

**Published:** 2023-03-23

**Authors:** Joshua Tam

**Affiliations:** ^1^Wellman Center for Photomedicine, Massachusetts General Hospital, Boston, MA, United States; ^2^Department of Dermatology, Harvard Medical School, Boston, MA, United States

**Keywords:** wound healing, volar skin, foot ulcer, keratinocytes, eccrine sweat gland, barrier function, specialization, palmoplantar

## Abstract

Foot ulcers, particularly in the diabetic setting, are a major medical and socioeconomic challenge. While the effects of diabetes and its various sequelae have been extensively studied, in the wound field it is commonly assumed that the wound healing process is essentially identical between different skin types, despite the many well-known specializations in palmoplantar skin, most of which are presumed to be evolutionary adaptations for weightbearing. This article will examine how these specializations could alter the wound healing trajectory and contribute to the pathology of foot ulcers.

## Introduction

Foot ulcers are a highly prevalent and costly medical burden. Diabetic foot ulcers (DFUs) are a particularly challenging wound type, with prevalence as high as 13% in North America (1), DFUs are the leading cause for lower-limb amputations worldwide (2), associated with 5-year mortality that exceeds many forms of cancer (3), and such high recurrence rates that leaders in the field have advocated for describing successful wound closures as “remission” rather than “cure” (4). There is clearly a pressing need to improve our understanding of DFU’s pathophysiology, to enable the development of more effective treatment options.

DFUs have a complex etiology with contributions from multiple disease factors, chief among them diabetes and its various detrimental consequences, as has been covered comprehensively in recent reviews on the topic (5, 6). DFUs commonly develop in the plantar metatarsal head and the heel, but this is typically attributed to increased tissue trauma related to weightbearing (7), rather than any inherent property of plantar skin tissue itself. More broadly, in the wound field there is a general presumption that the wound healing process is essentially homogenous all over the body, such that in *in vitro* studies skin cells’ anatomic origins are usually disregarded, in animal models the experimental wounds are almost exclusively produced on trunk/back skin, and in clinical studies biopsies from other anatomic areas are routinely used as controls to compare against plantar wound samples. Many of these are reasonable practices, especially in light of constraints associated with animal/human studies (e.g., animals’ trunk/back skin offers logistical benefits such as a large area and relatively flat contour, while requiring control wounds on the plantar skin in clinical studies is likely to be both impractical and ethically dubious), but they do carry the implicit, and largely unvalidated, assumption that results and conclusions drawn from wounds on any one part of the body are necessarily applicable to wounds occurring everywhere else. The body of data showing skin site-specific variations in cell populations (8–10) and wound healing (11) should cast doubt on this assumption, but it is especially questionable in palmoplantar skin, also known as volar skin, which has long been known for its highly specialized nature. This review aims to summarize the key volar-specific specializations that are likely to affect wound healing, and to make the case that these specializations should be taken into consideration both in our understanding of DFU’s pathophysiology, and in efforts to develop treatment options for DFU.

## Permeability barrier function

For terrestrial animals, limiting water loss is arguably the most crucial function of skin tissue (12). This role largely falls on the outermost *stratum corneum* of the epidermis, which is responsible for the bulk of skin’s permeability barrier function. The *stratum corneum* is many times thicker on volar skin than non-volar skin types (13), which may lead one to expect volar skin to have at least comparable, if not superior, barrier function. Surprisingly, multiple studies have shown that the volar *stratum corneum*’s barrier function is downright abysmal – the diffusion coefficient in the volar *stratum corneum* is about 100-fold higher than its non-volar counterparts (14, 15), and trans-epidermal water loss (TEWL) from the palms is about 4-fold higher than from most other parts of the body (15, 16). This poor barrier function in volar skin has potential implications for its healing response, as disruption of the skin barrier is thought to be the triggering event responsible for activating a cascade of downstream cellular and molecular responses that have been honed by millennia of evolutionary pressure toward restoring barrier function as quickly as possible (17), while continued deficiency in barrier function after wound closure is thought to be a major etiological driver behind hypertrophic scarring (one that can be alleviated by augmenting barrier function with occlusive dressings) (18). Thus the constitutively reduced barrier function in volar skin could have broad impacts on normal tissue homeostasis, initiation of the injury response, and the physiologic “end goal” for the repair process.

## Atypical structural and mechanical properties

As noted above, the volar stratum corneum is greatly thickened compared to non-volar skin. The same is true for all the suprabasal layers of the volar epidermis, such that according to one study the plantar epidermis is about 6-fold thicker than the epidermis on the dorsal foot (13). The greatly thickened volar epidermis may be expected to interfere with sensory function since mechanoreceptors in the skin are all located in the dermis, but it has been shown that foot callus thickness does not negatively affect sensitivity toward the types of tactile stimuli typically encountered during standing and walking (19, 20). This implies that volar skin must be stiff enough to transmit mechanical forces from the skin surface to the dermal mechanoreceptors with negligible dampening. Atomic force microscope (AFM) measurements have indeed confirmed that the stratum corneum, viable epidermis, and dermis in plantar skin having Young’s moduli that are 3-, 4.8- and 7.2-fold higher than their respective non-volar counterparts (21). Neither the cause of this increased stiffness, nor the extent to which it may be altered by injury/scarring are currently known – with the latter having obvious implications for post-healing tissue robustness and the risk of wound recurrence. The increased thickness and stiffness in volar skin may be advantageous for weightbearing, but are likely to negatively affect wound closure, as the physical contraction of wound edges is usually part of the wound closure mechanism, and increased tissue stiffness is likely to impede this contraction response, akin to the effects of wound splinting, which is a common method employed to delay healing in animal models (22). In addition to affecting the speed of acute wound closure, physical forces experienced by wound beds also have profound effects on long-term healing trajectory. This has long been known by surgeons who take pains to minimize transacting Langer lines when making skin incisions, and more recently the modulation of wound-associated mechanical forces, by either mechanical force redistribution or pharmaceutically interfering with mechanotransduction pathways, has been shown to substantially impact scar formation and tissue regeneration (23–25). Thus the rate and quality of wound healing in volar skin are both likely to be significantly affected by its unique mechanical environment, both intrinsically in the aforementioned increased tissue stiffness, and extrinsically where plantar skin in particular is routinely subjected to mechanical forces at magnitudes that are rarely ever experienced by other tissue/cell types.

## Glabrousness and the role of eccrine sweat glands

Thanks mostly to the advent of transgenic mouse models, recent years have seen an accumulation of a wealth of mechanistic understanding for the extensive contributions of hair follicles (HF), especially their resident stem/progenitor cell populations, to the wound healing process. However, this knowledge is not directly applicable to volar skin, which is completely devoid of pilosebaceous units, but is densely populated with eccrine sweat glands (ESGs). ESGs harbor their own stem cell populations that are also capable of participating in wound healing (26), but compared to HF-associated healing, our understanding of ESG-mediated healing is relatively light, mostly because mice, like most mammalian species, do not harbor ESGs in non-volar skin. Non-volar ESGs are a rare and relatively recent evolutionary development found only in catarrhine primates, and humans are more abundantly endowed with non-volar ESGs [which serve as our primary means for thermoregulation (27)] than any other animal, with 10x higher density of ESGs than our nearest primate relatives (28). Volar ESGs, on the other hand, are common to many mammalian species, where they have no meaningful role in thermoregulation, but instead modulate traction and are prominently featured in the “fight or flight” response (29, 30). In a study of human partial-thickness wound healing, it was shown that ESGs, rather than HFs, are the predominant contributor to the re-epithelialization process, in large part because ESGs outnumber HFs by a roughly 3:1 ratio in human non-volar skin (31). A related study also showed that aging caused a substantial reduction in ESG-associated post-injury epithelial outgrowth (due to loss of intercellular cohesiveness), while HF-associated outgrowth remained unchanged (32). This difference between ESG- and HF-derived outgrowth, if also true in volar ESGs (not a foregone conclusion given the aforementioned functional differences in volar vs. non-volar ESGs), suggests that the lack of hair in volar skin may render it uniquely vulnerable to aging-associated declines in healing capacity. Diabetes also has distinct effects on ESGs, with decreased sweating and the resultant dry skin being common manifestations of diabetic autonomic neuropathy (33). Whether this diminished functional state in diabetes-afflicted ESGs extends to their ability to contribute to wound healing is another as-yet unanswered question with profound implications for the healing capacity of DFUs.

## Unique and wound-like keratin composition

Keratins are the principal structural component of the epidermis, and the expression of Keratin 9 (K9) is arguably the most prominent molecular characteristic of volar keratinocytes (34–36), where, based on data from a knockout mouse model, K9 is required for terminal differentiation and mechanical integrity of the volar epidermis (37). It was previously thought that K9 expression was exclusive to volar skin, but recently K9 expression was also described in non-volar lesions of lichen simplex chronicus and psoriasis (36). Following a similar theme of shared features between volar and distressed skin, the volar epidermis has also been noted for expressing K6, 16, and 17 (38)–a trio of keratins whose expression in the interfollicular epidermis is otherwise characteristic of activated keratinocytes associated with wounds (38, 39) or pathologies such as psoriasis (40, 41). While K9 is expressed in both weightbearing and non-weightbearing areas of volar skin, the stress-response keratins K6 and 16 were found to be only expressed in the weightbearing heel, not in the non-weightbearing areas of the sole or the palm (42). Curiously, the same study showed upregulation of the basal keratins K5 and 14 (typically produced by keratinocytes in the mitotically active basal epidermis), and downregulation of the keratinocyte differentiation marker K10, also specifically in heel skin, suggesting that transitioning to a less-differentiated and wound-like phenotype may be involved in enabling volar keratinocytes to fulfill its weightbearing function. A natural question then is how these volar keratinocytes that are seemingly already in an activated, wound-like state at baseline would respond when faced with an actual injury. Would they be able to execute the wound repair process while remaining in their baseline state, without further activation? Would injury merely cause an increase in the same wound-associated phenotypes that are constitutively present in volar keratinocytes? Or are there alternative injury-response pathways that are specific to volar keratinocytes? The constitutive expression of certain wound-associated transcriptional networks is thought to prime the oral mucosa for rapid healing (43), whether the same is true for the wound-like volar keratinocyte phenotype remains to be determined, but given the existence of this phenotype, it seems prudent to reconsider the prevailing assumption that volar skin will necessarily respond to injuries in the same way as their non-volar counterparts.

## Fibroblast heterogeneity

Although historically (and at times still) treated as a homogenous and rather nondescript cell type, it is now abundantly clear that skin fibroblasts are composed of diverse subpopulations derived from different embryonic origins, with tremendous molecular and functional variability corresponding to both anatomic location and skin depth (8, 9, 44, 45). Different skin fibroblast subpopulations perform distinct functions in different phases of wound healing, and wound healing outcomes can be substantially altered by modulating specific fibroblast subpopulations (25, 46–48). It has long been known that volar fibroblasts harbor unique properties: classic heterotypic transplantation experiments have demonstrated that site-specific structural/morphologic characteristics of the epidermis are mostly determined by the origin of the underlying dermis (49), and subsequent studies have shown that paracrine signaling from volar fibroblasts can induce non-volar keratinocytes to produce the characteristic volar epidermal marker K9 (36, 50). The greatly increased stiffness of the volar dermis (21) also suggests likely differences in how volar fibroblasts construct and maintain the dermal architecture. DFU-associated volar fibroblasts are known to exhibit multiple functional alterations (51, 52), but beyond that the behavior/performance of volar fibroblasts in the wound setting remains largely unexplored.

## Immune system and its environment

The immune system plays a central role in orchestrating the wound healing process, and the failure of wound-associated inflammation to progress in an orderly manner is thought to be a major pathologic contributor to the chronicity of nonhealing wounds (53, 54). A key feature of the immune system is its high degree of specialization in different tissue sites (55), and there is evidence that innate and adaptive immunity both vary considerably between different skin areas (56). One of the main findings of a recent study utilizing bulk and single-cell RNA sequencing to compare human palm, sole, and hip skin was a broadly diminished immunological presence in palmoplantar skin, with reduced immune cell populations and downregulation of immunological processes (10). This is consistent with the reported 2–3 fold reduction in the density of Langerhans cells (LCs, specialized epidermal dendritic cells that serve as our foremost immunological barrier against the outside world) in normal, uninjured volar skin, compared to other non-volar skin areas (13, 57). While the exact role(s) of LCs in wound healing is still under investigation, increased LC in wound edges of DFUs has been correlated with improved healing rates (58), and the dynamics of epidermal LC population changes as well as expression of defensins have been found to diverge between plantar DFUs and venous ulcers on the calf in ways that suggest differing immune mechanisms between plantar and calf skin (57). It has also been shown that there is substantial region-specific heterogeneity in the skin microbiome, which is affected by local skin properties such as moisture and lipid content (59). The microbiome in plantar skin is likely subjected to additional influence from being the only skin area that is consistently in contact with the ground, and from the weightbearing areas of the feet having near neutral pH (60), whereas almost all other skin areas are normally slightly acidic, which is thought to serve a critical antimicrobial function (the so-called “acid mantle”) (61). Thus volar skin harbors distinctive features in both the intrinsic (immune system organization and functionality) and extrinsic (local microbiome) aspects of its immune milieu, which, given the crucial importance of the inflammatory response to the wound healing process, seem likely to impact wound healing in this tissue.

## Conclusion

**Figure 1:**
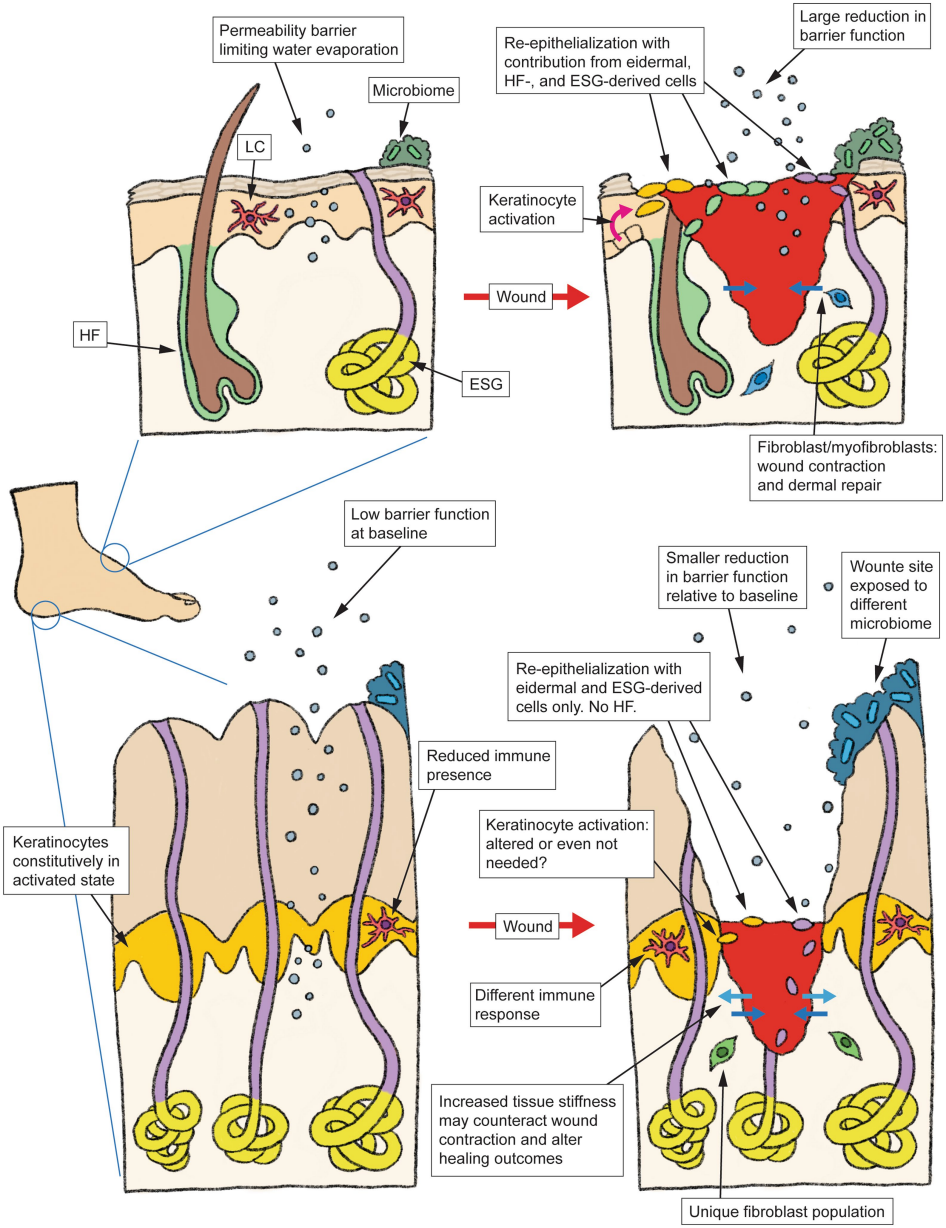
Schematic summarizing the anatomy and typical wound healing process in non-volar skin **(top)**, vs. the specialized architecture and physiological differences in volar skin **(bottom)** that may alter how this skin type responds to injury. HF, hair follicle, LC, Langerhans cell, ESG, eccrine sweat gland.

DFUs are well known to be a multifactorial disease, and the specialized physiology of volar skin could be one contributing factor that has been traditionally overlooked. Figure 1 summarizes some of the volar-specific skin properties that are likely to affect wound healing. The late Dr. Albert Kligman has observed that the stratum corneum of volar skin is “so unique as to require separate status” compared to its non-volar counterpart (62). With the body of evidence showing that the viable portions of volar skin are every bit as unique as its stratum corneum, it appears that more comprehensive investigations of the wound healing process specifically in volar skin are warranted. Our research group has recently begun such investigations (63), in hopes of shedding new light on the pathophysiology of foot ulcers.

## Author contributions

The author confirms being the sole contributor of this work and has approved it for publication.

## Conflict of interest

The author declares that the research was conducted in the absence of any commercial or financial relationships that could be construed as a potential conflict of interest.

The reviewer AV declared a shared affiliation with the author JT to the handling editor at the time of review.

## Publisher’s note

All claims expressed in this article are solely those of the authors and do not necessarily represent those of their affiliated organizations, or those of the publisher, the editors and the reviewers. Any product that may be evaluated in this article, or claim that may be made by its manufacturer, is not guaranteed or endorsed by the publisher.

## References

[1] Zhang P Lu J Jing Y Tang S Zhu D Bi Y. Global epidemiology of diabetic foot ulceration: a systematic review and meta-analysis (dagger). Ann Med. (2017) 49:106–16. doi: 10.1080/07853890.2016.1231932, PMID: 27585063

[2] Bernatchez J Mayo A Kayssi A. The epidemiology of lower extremity amputations, strategies for amputation prevention, and the importance of patient-centered care. Semin Vasc Surg. (2021) 34:54–8. doi: 10.1053/j.semvascsurg.2021.02.011, PMID: 33757636

[3] Armstrong DG Swerdlow MA Armstrong AA Conte MS Padula WV Bus SA. Five year mortality and direct costs of care for people with diabetic foot complications are comparable to cancer. J Foot Ankle Res. (2020) 13:16. doi: 10.1186/s13047-020-00383-2, PMID: 32209136 PMC7092527

[4] Armstrong DG Mills JL. Toward a change in syntax in diabetic foot care: prevention equals remission. J Am Podiatr Med Assoc. (2013) 103:161–2. doi: 10.7547/1030161, PMID: 23536510

[5] Armstrong DG Boulton AJM Bus SA. Diabetic foot ulcers and their recurrence. N Engl J Med. (2017) 376:2367–75. doi: 10.1056/NEJMra1615439 28614678

[6] Burgess JL Wyant WA Abdo Abujamra B Kirsner RS Jozic I. Diabetic wound-healing science. Lietuvis̆koji Med. (2021) 57. doi: 10.3390/medicina57101072, PMID: 34684109 PMC8539411

[7] Oliver TI Mutluoglu M. Diabetic Foot Ulcer. Treasure Island, FL: StatPearls Publishing (2022). 30726013

[8] Chang HY Chi JT Dudoit S Bondre C van de Rijn M Botstein D. Diversity, topographic differentiation, and positional memory in human fibroblasts. Proc Natl Acad Sci U S A. (2002) 99:12877–82. doi: 10.1073/pnas.162488599, PMID: 12297622 PMC130553

[9] Rinn JL Bondre C Gladstone HB Brown PO Chang HY. Anatomic demarcation by positional variation in fibroblast gene expression programs. PLoS Genet. (2006) 2:e119. doi: 10.1371/journal.pgen.0020119, PMID: 16895450 PMC1523235

[10] Wiedemann J Billi AC Bocci F Kashgari G Xing E Tsoi LC. Differential cell composition and split epidermal differentiation in human palm, sole, and hip skin. Cell Rep. (2023) 42:111994. doi: 10.1016/j.celrep.2023.111994, PMID: 36732947 PMC9939370

[11] Usansky I Jaworska P Asti L Kenny FN Hobbs C Sofra V. A developmental basis for the anatomical diversity of dermis in homeostasis and wound repair. J Pathol. (2021) 253:315–25. doi: 10.1002/path.5589, PMID: 33197044 PMC7898902

[12] Madison KC. Barrier function of the skin: "la raison d'etre" of the epidermis. J Invest Dermatol. (2003) 121:231–41. doi: 10.1046/j.1523-1747.2003.12359.x, PMID: 12880413

[13] Vela-Romera A Carriel V Martín-Piedra MA Aneiros-Fernández J Campos F Chato-Astrain J. Characterization of the human ridged and non-ridged skin: a comprehensive histological, histochemical and immunohistochemical analysis. Histochem Cell Biol. (2019) 151:57–73. doi: 10.1007/s00418-018-1701-x, PMID: 30099600 PMC6328512

[14] Blank IH. Further observations on factors which influence the water content of the stratum corneum. J Invest Dermatol. (1953) 21:259–71. doi: 10.1038/jid.1953.100, PMID: 13096868

[15] Baker H Kligman AM. Measurement of transepidermal water loss by electrical hygrometry. Instrumentation and responses to physical and chemical insults. Arch Dermatol. (1967) 96:441–52. doi: 10.1001/archderm.1967.01610040091018, PMID: 6046392

[16] Kleesz P Darlenski R Fluhr JW. Full-body skin mapping for six biophysical parameters: baseline values at 16 anatomical sites in 125 human subjects. Skin Pharmacol Physiol. (2012) 25:25–33. doi: 10.1159/000330721, PMID: 21912200

[17] Wikramanayake TC Stojadinovic O Tomic-Canic M. Epidermal differentiation in barrier maintenance and wound healing. Adv Wound Care. (2014) 3:272–80. doi: 10.1089/wound.2013.0503, PMID: 24669361 PMC3955965

[18] O'Shaughnessy KD De La Garza M Roy NK Mustoe TA. Homeostasis of the epidermal barrier layer: a theory of how occlusion reduces hypertrophic scarring. Wound Repair Regen. (2009) 17:700–8. doi: 10.1111/j.1524-475X.2009.00534.x, PMID: 19769722

[19] Holowka NB Wynands B Drechsel TJ Yegian AK Tobolsky VA Okutoyi P. Foot callus thickness does not trade off protection for tactile sensitivity during walking. Nature. (2019) 571:261–4. doi: 10.1038/s41586-019-1345-6 31243365

[20] Wynands B Zippenfennig C Holowka NB Lieberman DE Milani TL. Does plantar skin abrasion affect cutaneous mechanosensation? Physiol Rep. (2022) 10:e15479. doi: 10.14814/phy2.15479, PMID: 36259120 PMC9579735

[21] Boyle CJ Plotczyk M Villalta SF Patel S Hettiaratchy S Masouros SD. Morphology and composition play distinct and complementary roles in the tolerance of plantar skin to mechanical load. Sci Adv. (2019) 5:eaay0244. doi: 10.1126/sciadv.aay0244, PMID: 31633031 PMC6785259

[22] Galiano RD Michaels, V J Dobryansky M Levine JP Gurtner GC. Quantitative and reproducible murine model of excisional wound healing. Wound Repair Regen. (2004) 12:485–92. doi: 10.1111/j.1067-1927.2004.12404.x, PMID: 15260814

[23] Fu S Panayi A Fan J Mayer HF Daya M Khouri RK. Mechanotransduction in wound healing: from the cellular and molecular level to the clinic. Adv Skin Wound Care. (2021) 34:67–74. doi: 10.1097/01.ASW.0000725220.92976.a7, PMID: 33443911

[24] Kuehlmann B Bonham CA Zucal I Prantl L Gurtner GC. Mechanotransduction in wound healing and fibrosis. J Clin Med. (2020) 9:1423. doi: 10.3390/jcm9051423, PMID: 32403382 PMC7290354

[25] Mascharak S desJardins-Park HE Davitt MF Griffin M Borrelli MR Moore AL. Preventing Engrailed-1 activation in fibroblasts yields wound regeneration without scarring. Science. (2021) 372:eaba2374. doi: 10.1126/science.aba2374, PMID: 33888614 PMC9008875

[26] Lu CP Polak L Rocha AS Pasolli HA Chen SC Sharma N. Identification of stem cell populations in sweat glands and ducts reveals roles in homeostasis and wound repair. Cells. (2012) 150:136–50. doi: 10.1016/j.cell.2012.04.045, PMID: 22770217 PMC3423199

[27] Folk GE Jr Semken HA Jr. The evolution of sweat glands. Int J Biometeorol. (1991) 35:180–6. doi: 10.1007/BF01049065 1778649

[28] Kamberov YG Guhan SM DeMarchis A Jiang J Wright SS Morgan BA. Comparative evidence for the independent evolution of hair and sweat gland traits in primates. J Hum Evol. (2018) 125:99–105. doi: 10.1016/j.jhevol.2018.10.008, PMID: 30502901 PMC6289065

[29] Adelman S Taylor CR Heglund NC. Sweating on paws and palms: what is its function? Am J Phys. (1975) 229:1400–2. doi: 10.1152/ajplegacy.1975.229.5.1400, PMID: 1200160

[30] Taylor NA Machado-Moreira CA. Regional variations in transepidermal water loss, eccrine sweat gland density, sweat secretion rates and electrolyte composition in resting and exercising humans. Extrem Physiol Med. (2013) 2:4. doi: 10.1186/2046-7648-2-4, PMID: 23849497 PMC3710196

[31] Rittie L Sachs DL Orringer JS Voorhees JJ Fisher GJ. Eccrine sweat glands are major contributors to reepithelialization of human wounds. Am J Pathol. (2013) 182:163–71. doi: 10.1016/j.ajpath.2012.09.019, PMID: 23159944 PMC3538027

[32] Rittie L Farr EA Orringer JS Voorhees JJ Fisher GJ. Reduced cell cohesiveness of outgrowths from eccrine sweat glands delays wound closure in elderly skin. Aging Cell. (2016) 15:842–52. doi: 10.1111/acel.12493, PMID: 27184009 PMC5013029

[33] Vinik AI Maser RE Mitchell BD Freeman R. Diabetic autonomic neuropathy. Diabetes Care. (2003) 26:1553–79. doi: 10.2337/diacare.26.5.1553 12716821

[34] Moll R Franke WW Schiller DL Geiger B Krepler R. The catalog of human cytokeratins: patterns of expression in normal epithelia, tumors and cultured cells. Cells. (1982) 31:11–24. doi: 10.1016/0092-8674(82)90400-7, PMID: 6186379

[35] Knapp AC Heid H Franke WW Hatzfeld M Jorcano JL Moll R. Cytokeratin no. 9, an epidermal type I keratin characteristic of a special program of keratinocyte differentiation displaying body site specificity. J Cell Biol. (1986) 103:657–67. doi: 10.1083/jcb.103.2.657, PMID: 2426283 PMC2113844

[36] Kim D Hossain MZ Nieves A Gu L Ratliff TS Mi Oh S. To control site-specific skin gene expression, autocrine mimics paracrine canonical Wnt signaling and is activated ectopically in skin disease. Am J Pathol. (2016) 186:1140–50. doi: 10.1016/j.ajpath.2015.12.030, PMID: 27105735 PMC4861769

[37] Fu DJ Thomson C Lunny DP Dopping-Hepenstal PJ McGrath JA Smith FJD. Keratin 9 is required for the structural integrity and terminal differentiation of the palmoplantar epidermis. J Invest Dermatol. (2014) 134:754–63. doi: 10.1038/jid.2013.356, PMID: 23962810 PMC3923277

[38] Freedberg IM Tomic-Canic M Komine M Blumenberg M. Keratins and the keratinocyte activation cycle. J Invest Dermatol. (2001) 116:633–40. doi: 10.1046/j.1523-1747.2001.01327.x 11348449

[39] Paladini RD Takahashi K Bravo NS Coulombe PA. Onset of re-epithelialization after skin injury correlates with a reorganization of keratin filaments in wound edge keratinocytes: defining a potential role for keratin 16. J Cell Biol. (1996) 132:381–97. doi: 10.1083/jcb.132.3.381, PMID: 8636216 PMC2120730

[40] Leigh IM Navsaria H Purkis PE Mckay IA Bowden PE Riddle PN. Keratins (K16 and K17) as markers of keratinocyte hyperproliferation in psoriasis in vivo and in vitro. Br J Dermatol. (1995) 133:501–11. doi: 10.1111/j.1365-2133.1995.tb02696.x, PMID: 7577575

[41] Yang L Fan X Cui T Dang E Wang G. Nrf2 promotes keratinocyte proliferation in psoriasis through up-regulation of keratin 6, keratin 16, and keratin 17. J Invest Dermatol. (2017) 137:2168–76. doi: 10.1016/j.jid.2017.05.015, PMID: 28576737

[42] Egelrud T Stigbrand T. Regional variations in cytokeratin expression in palmo-plantar epidermis. Acta Derm Venereol. (1989) 69:373–9. 2477975

[43] Iglesias-Bartolome R Uchiyama A Molinolo AA Abusleme L Brooks SR Callejas-Valera JL. Transcriptional signature primes human oral mucosa for rapid wound healing. Sci Transl Med. (2018) 10. doi: 10.1126/scitranslmed.aap8798, PMID: 30045979 PMC6598699

[44] Sorrell JM Caplan AI. Fibroblasts-a diverse population at the center of it all. Int Rev Cell Mol Biol. (2009) 276:161–214. doi: 10.1016/S1937-6448(09)76004-6 19584013

[45] Driskell RR Watt FM. Understanding fibroblast heterogeneity in the skin. Trends Cell Biol. (2015) 25:92–9. doi: 10.1016/j.tcb.2014.10.001 25455110

[46] Driskell RR Lichtenberger BM Hoste E Kretzschmar K Simons BD Charalambous M. Distinct fibroblast lineages determine dermal architecture in skin development and repair. Nature. (2013) 504:277–81. doi: 10.1038/nature12783, PMID: 24336287 PMC3868929

[47] Jiang D Correa-Gallegos D Christ S Stefanska A Liu J Ramesh P. Two succeeding fibroblastic lineages drive dermal development and the transition from regeneration to scarring. Nat Cell Biol. (2018) 20:422–31. doi: 10.1038/s41556-018-0073-8, PMID: 29593327

[48] Sinha S Sparks HD Labit E Robbins HN Gowing K Jaffer A. Fibroblast inflammatory priming determines regenerative versus fibrotic skin repair in reindeer. Cells. (2022) 185:4717–4736.e25. doi: 10.1016/j.cell.2022.11.004, PMID: 36493752 PMC9888357

[49] Billingham RE Silvers WK. Studies on the conservation of epidermal specificies of skin and certain mucosas in adult mammals. J Exp Med. (1967) 125:429–46. doi: 10.1084/jem.125.3.429, PMID: 5334545 PMC2138290

[50] Yamaguchi Y Itami S Tarutani M Hosokawa K Miura H Yoshikawa K. Regulation of keratin 9 in nonpalmoplantar keratinocytes by palmoplantar fibroblasts through epithelial-mesenchymal interactions. J Invest Dermatol. (1999) 112:483–8. doi: 10.1046/j.1523-1747.1999.00544.x, PMID: 10201533

[51] Loots MAM Kenter SB Au FL van Galen WJM Middelkoop E Bos JD. Fibroblasts derived from chronic diabetic ulcers differ in their response to stimulation with EGF, IGF-I, bFGF and PDGF-AB compared to controls. Eur J Cell Biol. (2002) 81:153–60. doi: 10.1078/0171-9335-00228, PMID: 11998867

[52] Maione AG Smith A Kashpur O Yanez V Knight E Mooney DJ. Altered ECM deposition by diabetic foot ulcer-derived fibroblasts implicates fibronectin in chronic wound repair. Wound Repair Regen. (2016) 24:630–43. doi: 10.1111/wrr.12437, PMID: 27102877 PMC5500637

[53] Pastar I Balukoff NC Marjanovic J Chen VY Stone RC Tomic-Canic M. Molecular pathophysiology of chronic wounds: current state and future directions. Cold Spring Harb Perspect Biol. (2022) 2022:a041243. doi: 10.1101/cshperspect.a041243, PMID: 36123031 PMC10024648

[54] Larouche J Sheoran S Maruyama K Martino MM. Immune regulation of skin wound healing: mechanisms and novel therapeutic targets. Adv Wound Care. (2018) 7:209–31. doi: 10.1089/wound.2017.0761, PMID: 29984112 PMC6032665

[55] Poon MML Farber DL. The whole body as the system in systems immunology. iScience. (2020) 23:101509. doi: 10.1016/j.isci.2020.101509, PMID: 32920485 PMC7491152

[56] Béke G Dajnoki Z Kapitány A Gáspár K Medgyesi B Póliska S. Immunotopographical differences of human skin. Front Immunol. (2018) 9:424. doi: 10.3389/fimmu.2018.00424, PMID: 29556238 PMC5844973

[57] Galkowska H Olszewski WL Wojewodzka U. Expression of natural antimicrobial peptide beta-defensin-2 and Langerhans cell accumulation in epidermis from human non-healing leg ulcers. Folia Histochem Cytobiol. (2005) 43:133–6. 16201312

[58] Stojadinovic O Yin N Lehmann J Pastar I Kirsner RS Tomic-Canic M. Increased number of Langerhans cells in the epidermis of diabetic foot ulcers correlates with healing outcome. Immunol Res. (2013) 57:222–8. doi: 10.1007/s12026-013-8474-z, PMID: 24277309 PMC4349345

[59] Grice EA Kong HH Conlan S Deming CB Davis J Young AC. Topographical and temporal diversity of the human skin microbiome. Science. (2009) 324:1190–2. doi: 10.1126/science.1171700, PMID: 19478181 PMC2805064

[60] Jolly HW Jr Hailey CW Netick J pH determinations of the skin. Readings under normal and abnormal conditions. J Invest Dermatol. (1961) 36:305–8. doi: 10.1038/jid.1961.51 13790435

[61] Surber C Humbert P Abels C Maibach H. The acid mantle: a myth or an essential part of skin health? Curr Probl Dermatol. (2018) 54:1–10. doi: 10.1159/000489512 30125885

[62] Kligman AM. The biology of the stratum corneum In: Montagna W Lobitz WB, editors. The Epidermis. Cambridge: Academic Press (1964). 387–433.

[63] Stalnaker KJ Fuchs C Slate A Camacho JN Pham L Wang Y. Boot camp: training and dressing regimens for modeling plantar wounds in the swine. Lab Anim. (2022) 57:59–68. doi: 10.1177/00236772221111058, PMID: 35962527

